# FANTOM5 transcriptome catalog of cellular states based on Semantic MediaWiki

**DOI:** 10.1093/database/baw105

**Published:** 2016-07-09

**Authors:** Imad Abugessaisa, Hisashi Shimoji, Serkan Sahin, Atsushi Kondo, Jayson Harshbarger, Marina Lizio, Yoshihide Hayashizaki, Piero Carninci, The FANTOM consortium, Alistair Forrest, Takeya Kasukawa, Hideya Kawaji

**Affiliations:** ^1^Division of Genomic Technologies (DGT), RIKEN Center for Life Science Technologies (CLST), Kanagawa 230-0045, Japan; ^2^RIKEN Omics Science Center (OSC), 1-7-22 Suehiro-cho, Tsurumi-ku, Yokohama 230-0045, Japan^‡^; ^3^RIKEN Preventive Medicine and Diagnosis Innovation Program, Wako, Saitama 351-0198, Japan; ^4^Harry Perkins Institute of Medical Research, QEII Medical Centre and Centre for Medical Research, the University of Western Australia, Nedlands, Western Australia, Australia; ^5^Preventive Medicine and Applied Genomics Unit, RIKEN Advanced Center for Computing and Communication, Kanagawa 230-0045, Japan

## Abstract

The Functional Annotation of the Mammalian Genome project (FANTOM5) mapped transcription start sites (TSSs) and measured their activities in a diverse range of biological samples. The FANTOM5 project generated a large data set; including detailed information about the profiled samples, the uncovered TSSs at high base-pair resolution on the genome, their transcriptional initiation activities, and further information of transcriptional regulation. Data sets to explore transcriptome in individual cellular states encoded in the mammalian genomes have been enriched by a series of additional analysis, based on the raw experimental data, along with the progress of the research activities. To make the heterogeneous data set accessible and useful for investigators, we developed a web-based database called Semantic catalog of Samples, Transcription initiation And Regulators (SSTAR). SSTAR utilizes the open source wiki software MediaWiki along with the Semantic MediaWiki (SMW) extension, which provides flexibility to model, store, and display a series of data sets produced during the course of the FANTOM5 project. Our use of SMW demonstrates the utility of the framework for dissemination of large-scale analysis results. SSTAR is a case study in handling biological data generated from a large-scale research project in terms of maintenance and growth alongside research activities.

**Database URL:** http://fantom.gsc.riken.jp/5/sstar/

## Introduction

Recent developments in sequencing technology and computational methods have influenced the way molecular biology research is conducted and enables the identification and profiling of molecules at a very high resolution with high accuracy (1). In the field of transcriptomics, the presence of RNA molecules was characterized by sequencing expressed sequence tags (2–4), and their relative abundance was quantified by microarray in a high-throughput manner based on predesigned probes (5). The emergence of next-generation sequencers enabled researchers to quantify transcript structure [exon structure by RNA-Seq (6)] and genome-wide transcription initiation sites (cap analysis of gene expression, CAGE) (7) without previous knowledge of individual transcripts. To ensure reproducibility and increase the utility of high-throughput data, public repositories have been established and maintained. For example the International Nucleotide Sequence Database Collaboration for sequence data (8), NCBI GEO (9), EMBL ArrayExpress (10) for gene expression data and SRA/EGA/DRA for next-generation sequencing (11). On top of these data repositories, targeted databases have been developed that provide curated data to facilitate biological interpretation and findings, such as the Gene Expression Atlas (12), BioGPS (13), UCSC Genome Browser (14), FANTOM3 CAGE databases (15) and FANTOM4 (16).

The FANTOM5 project aimed to obtain transcriptome maps of mammalian genomes in a comprehensive set of cellular states. In particular, human primary cells are of major focus since they have been poorly surveyed with genome-wide methods due to limitation of sample availabilities. The project has identified transcription start sites (TSSs) and measured TSS activities in a diverse range of samples (∼1800 for human and ∼1000 for mouse) using a CAGE method adapted to a single molecule sequencer (17). The analysis results in FANTOM5 range from genomic information, like the definition of TSS regions and their association with known genes (primary analysis), to higher-level analysis, including co-expression clustering of TSSs, statistical assessment of transcription-factor-binding-site motifs within CAGE peaks, samples, and enrichment analysis of pathways or samples. The selection of samples, which covers cell lines, tissues, and primary cells, provided a rich opportunity to explore transcriptome states encoded in the genome. In order to classify these samples, the FANTOM5 sample ontology (FF ontology) was developed consisting of multiple subclasses representing distinct aspects of the samples, such as cell types, anatomical tissues, and diseases (18). In handling these data sets, we faced two major challenges besides increasing data sizes: adaptation to new types of data being generated as research grows, and flexible representation of associations across heterogeneous data. Research activities generate novel ideas, which in turn generate new data. New data do not necessarily fit to existing data models, and its adaptation often requires schematic changes. In parallel to the schematic changes to the data model, visual representation has to be designed for manual inspection. Besides additional representation for newly produced data, its association has to be shown also in the representation of existing data. It requires incremental changes, which can be effectively assisted by flexibility in data representation.

Here, we present a web-based database called Semantic catalog of Samples, Transcription initiation And Regulators (SSTAR) as a platform to deliver FANTOM5 sample information and analysis results to the research community. We employed Semantic MediaWiki (SMW), the open source wiki engine developed for Wikipedia with extended capacity to store additional data (termed semantic properties) alongside wiki content (19). Queries on semantic properties, termed semantic query, improve upon traditional keyword search. The semantic query can be embedded in wiki pages to show the query result in-line with wiki context (termed in-line semantic query). We used semantic properties to adopt new data without destructive schematic change on existing data, and in-line semantic query for representations of data associations. We added several visual components for genomic view, quantitative value display, and ontology classes through development of our custom extension (SSTAR extension). In the course of the FANTOM5 project (17), several database systems have been developed, such as the ZENBU data visualization platform offering all functionalities of a genome browser with interactive operations (20), the FANTOM5 Table Extraction Tool enabling us efficiently to extract subsets of data from selected FANTOM5 data files, and a BioMart (21) instance enabling us to obtain subsets of FANTOM5 promoters and samples via a well-known interface. Although these systems are designed for specific data sets or functionalities, SSTAR is developed to support heterogeneous data, including novel type of data requiring complex associations across the heterogeneous data types. All of these systems are complementary, and several use cases are described in (18). For more information about FANTOM5 data, systems, publications and context, see http://fantom.gsc.riken.jp/5/.

## Materials and Methods

### Data sets in FANTOM5 SSTAR

**Figure 1.**
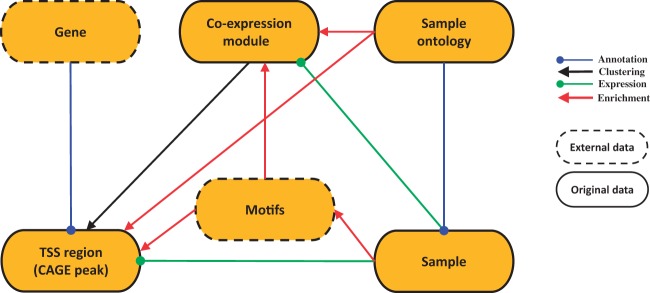
SSTAR data model. SSTAR data model consists of six classes, those represents the main ‘categories’ in SMW. The oval represents a class and the kind of the data stored. Relationship between any two categories is represented as an arrow. The direction of the arrow indicates which of the two classes stores the relationship (indicated by the end of the arrow) as a class attribute. The head and color of the arrow indicates the type of relationship.

SSTAR stores and manages two kinds of data sets: original (raw and processed) data generated by the FANTOM5 consortium and external data supporting interpretation of the FANTOM5 data (Figure 1). The original raw data set consists of sample RNA and sequencing library metadata in sample and data relationship format (SDRF), sequence data in the FASTQ format, genome mapping data in the BAM format and CTSS (CAGE tag starting site) profiles in the BED format. The raw data had been deposited to DDBJ DRA under accession number DRA000991, DRA001026, DRA001027, DRA001028, DRA001101, DRA002216, DRA002711, DRA002747, DRA002748 (17, 22).

The original processed data contains a series of transcriptional initiation profiles based on the CAGE technology and the decomposition-based peak identification method (17). We used 201 802 and 158 966 robust CAGE peaks (>3 copies per TSS), as transcription initiation regions in human and mouse genomes, respectively. Individual CAGE peaks were associated with genes based on their genomic coordinates, and activity (or expression) levels of the CAGE peaks were quantified according to the read counts in the CAGE profiles by the FANTOM5 consortium. CAGE peaks with similar expression profiles were also grouped into gene co-expression modules by applying the Markov Cluster Algorithm (23) to the CAGE peak expression data set by the consortium. All the process data are available at FANTOM5 web site (http://fantom.gsc.riken.jp/5/datafiles/).

The FF ontology is composed of three ontologies: cell type ontology (CL), cross-species anatomical ontology (UBERON), and disease ontology. The FF ontology files are accessible from the GitHub repository (https://github.com/cmungall/fantom5-ontology) in Ontology Web Language (OWL) format.

Data sets from external sources include, but are not limited to, gene models retrieved from Entrez Gene in NCBI and information on reported motifs (DNA patterns bound by transcription factors) retrieved from JASPAR (24).

### Data storage

**Figure 2.**
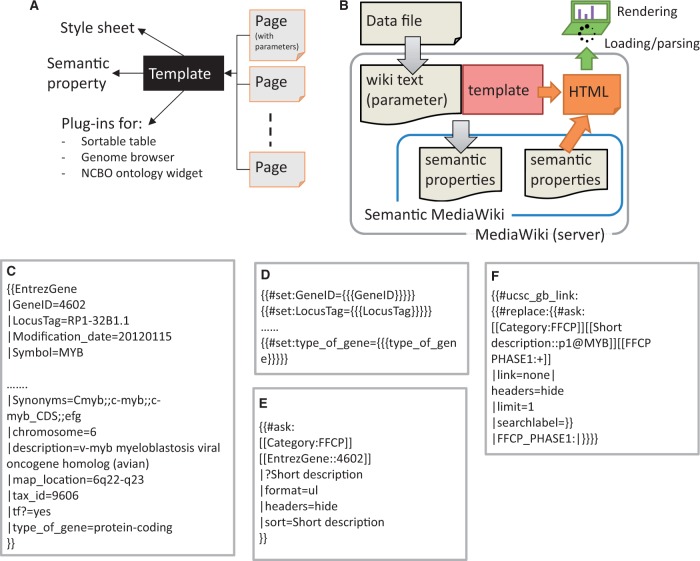
Implementation scheme of data model with SMW template. **(A)** Template dependencies. Each page in SSTAR use one or more template from SMW, the template points to different style sheets, calls semantic property and SSTAR plug-ins to deliver particular function. **(B)** Data flow from depositing the data file in MediaWiki server to render the page into the client. **(C)** Code snippet showing template call (EntrezGene) with the semantic properties to generate the page with EntrezGene:4602 http://fantom.gsc.riken.jp/5/sstar/EntrezGene:4602. **(D)** Statements in the ‘EntrezGene’ template to store template parameters as semantic properties. The statements will add the semantic properties ‘GeneID’, ‘LocusTag’ and ‘type_of_gene’. **(E)** An example of inline semantic query, retrieving the association between two categories (gene and CAGE peaks) and show the result in an unnumbered list. **(F)** An example of the inline semantic query modified to call SSTAR ucsc_gb_link function to provide the genomic view of the FFCP in UCSC genome browser.

The MediaWiki system stores the majority of data as Wiki markup text. It also supports a template scheme to specify presentation style. We used the template parameters to store data as semantic properties (see below), in addition to descriptive text (Figure 2A and B). The templates are used to specify a graphical layout and to set and query semantic properties. The process to generate semantic properties in a SWM system consists of two consecutive steps: (i) all data objects are imported as wiki markup (see ‘Data modeling and implementation of heterogeneous and complex biological states in SMW’ in the ‘Results and Discussion’ section), (ii) the imported markup text is then parsed by the SMW system to generate semantic properties.

### Interface to the end users

The SSTAR landing page summarizes the content of FANTOM5 data sets into categories: human data, mouse data, cross-species data and others (main menu and submenus are shown in Supplementary Figure S1).

**Figure 3.**
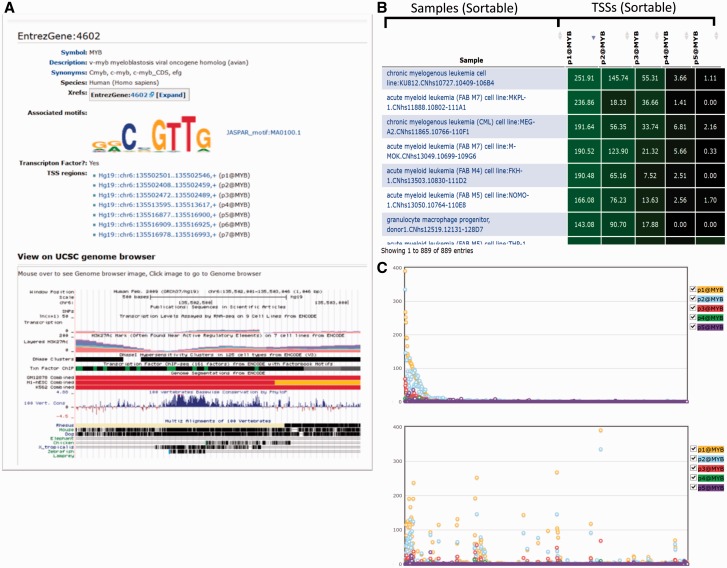
Graphical representation of a gene (MYB). **(A)** Result of the search for MYB gene in SSTAR, with its associated Motifs and list of TSS regions. The user is able to get UCSC genome browser view of the MYB gene. **(B)** The table shows the expression of five TSS regions associated with MYB. **(C)** The graphical representation of the B) in which X-axis represents individual samples and y-axis represents expression intensities.

In addition to the standard layouts in MediaWiki we enhanced the function of data tables in the SSTAR system to enable search, export, and data plotting using various JavaScript libraries. We further added components to support specific visualizations for the FANTOM5 data set such as a genomic view with UCSC genome browser (Figure 3A) and a tree view of the ontology structure with NCBO’s ontology visualization widget (http://www.bioontology.org/wiki/index.php/NCBO_Widgets#Ontology_visualization_widget; Supplementary Figure S2C). These functions are implemented as custom extensions to MediaWiki, which calls external services or API (Application Program Interface) provided outside of SSTAR.

### Performance testing

We measured the time to complete a user’s request, which consists of the time for the server to start its response (termed ‘latency’), and for a client to load, parse and render the data in a page (termed ‘loading and parsing’). We randomly selected 1% of the total number of the pages in each of the main categories (in Figure 1) to measure distribution of required time per page [4179 pages in total; see Supplementary Table S1 for a summary and the full list is available at our web site (http://fantom.gsc.riken.jp/5/suppl/Abugessaisa_et_al_2016/)]. We also evaluated the effect of server side memory caching (Memcached, http://www.mediawiki.org/wiki/Manual:Configuration_settings#Memcached_settings). The detailed description of the performance evaluation method is in the Supplementary Materials.

## Results and Discussion

### Data modeling and implementation of heterogeneous and complex biological states in SMW

We have six data classes corresponding to FANTOM5 data types (Figure 1**)**: genes, TSS regions (CAGE peaks), co-expression modules of similarly activated TSS regions, samples, FF ontology terms, and motifs. The six classes are interconnected through different kinds of relationships: associations, annotations, clustering, expression and enrichment. The number of data objects in each class and their properties are summarized in Table 1.

**Table 1.** Summary of the number of objects in SSTAR

Data class > Category	Attributes	**Data objects or attributes**	
		human	mouse
***TSS region > FFCP***	human readable description	201 802	158 966
	association with genes^a^	174 802	136 492
	co-expression module^a^	240 776	180 000
	CAGE expression^a^	201 802	158 966
	ontology-based sample term enrichment analysis^a^	6 097 409	2 843 664
***Gene > EntrezGene***	human readable description	408 504	317 946
	DNA-binding motifs (only for transcription factors)	91	88
	association with TSS regions (CAGE peaks)+	174 802	136 492
***Co-expression module> Coexpression_cluster, MCL_coexpression_mm9***	TSS region (CAGE peak) cluster+	310	278
	pathway enrichment	1672	1385
	gene ontology enrichment	48 473	38 751
	ENCODE TF ChIP-seq peak enrichment analysis (with Coexpression)	29 778	N/A
	sample ontology enrichment^a^	224 852	87 181
	relative expression of the co-expression cluster^a^	889	389
***Sample > FF Sample***	human readable description	1816	1 018
	classification according to the sample ontology (‘Ancestor terms’)^a^	34 689	12 357
	transcription factors with enriched expression	983 000	367 000
	co-expression clusters with enriched expression	300 798	81 474
	repeat families with enriched expression	130 789	34 865
	overrepresented JASPAR motifs	112	112
	overrepresented novel unique motifs	169	168
	Homer de novo motifs	39 320	14 680
***Sample ontology > FF ontology***	description	3782	
	parent terms	9640	
	children terms	9620	
***Motifs > JASPER_motif, Novel_motif***	human readable description	687	
	association to promoter expression^a^	1278	

SSTAR data objects and their corresponding categories in MediaWiki and the attributes in each object. Relationship to other objects are indicated with ^a^ (*:forward and +reverse).

In our implementation we used the MediaWiki concepts of ‘category’, ‘page’ and ‘template parameter’ for data class, data object, and object attributes respectively. The attributes were handled as semantic properties (Table 2). Figure 2A and B shows our scheme of storing object attributes as template parameters, and subsequently as semantic properties. Figure 2C–E illustrates individual steps to store and retrieve attributes based on a gene MYB as an example. Attributes of the gene are described as parameters of a template ‘EntrezGene’ in a page of ‘Gene’ category (Figure 2C), and passed to semantic properties using the no. set function within the template (Figure 2D). The properties can be retrieved by using a semantic query (Figure 2E).

**Table 2.** Mapping of the modeling entities in SSTAR

Modeling entities	MediaWiki	SMW
Class	Category	
Object	Page	
Attributes	Template parameters	Semantic properties

Data objects and their relationships ; the table show the mapping between the data model (column 1), MediaWiki(column 2) and the SMW (column 3).

Associations between data objects (pages) are essential to represent the data models as well as to navigate across multiple pages. We stored these associations as semantic properties**.** While they are stored in only one of the data objects (pages), they can be retrieved immediately in the other object by using semantic queries (Figure 2E). For example, when one gene is activated by multiple promoters it can be associated with multiple CAGE peaks. The number of associated CAGE peaks is dependent on how complex the regulation is that drives the gene. Although we stored associations between gene and CAGE peaks in the ‘CAGE peak’ pages only, we can show ‘associated CAGE peaks’ by writing an inline semantic query (Figure 2E) in “gene” pages (Figure 3A, TSS regions section). This structure was used for all associations illustrated in Figure 1. Semantic queries are not limited for use within SSTAR. Users and external programs can access SSTAR semantic properties by accessing the semantic query interface (http://fantom.gsc.riken.jp/5/sstar/Special:Ask).

### Evolution of views

We configured all pages based on the templates as described, and the templates are shared among pages to provide consistent interfaces for data objects in the same class. A template is simply an editable page equivalent to other wiki pages. All of its contents, including presented data, semantic query and graphical layout, can be updated rapidly as research activities move forward.

For example, in the FANTOM5 project, many data sets are usually presented as a table. Many pages display one or multiple data tables. A gene page, e.g. displays an expression table of CAGE peaks associated with the gene, whereas a sample page displays tables of highly expressed CAGE peaks of transcription factors in the sample, co-expressed clusters with enriched expression in the sample, known TFBS (DNA) motifs significantly associated with promoter activities, and novel TFBS motifs discovered in the proximal region of promoters active in this sample. We initially provided these data sets as simple tables; however, it became inefficient for investigators to inspect a fixed table consisting of hundreds of rows without an interactive interface. In order to improve navigation of data tables in SSTAR, we employed a JavaScript module (FLOT, http://www.flotcharts.org/) that enables dynamic operations on the tables **(**Figure 3B and C and Supplementary Figure S3). The table in Figure 3B shows activities (expressions) of five TSS regions associated with MYB, in individual samples, and Figure 3C is its graphical representation, where the *x*-axis represents individual samples and the *y*-axis represents expression intensities of TSS regions (represented by different colors). The table is sortable and linked with the graphical representation, such that when users modify the sort order, changes are immediately reflected in the visualization. Furthermore, when a user clicks a point in the chart, its corresponding CAGE peak is indicated in the table. This enhancement is implemented by modification of the template page, which demonstrates that the SMW framework allowed us to evolve the page layout the needs of researchers’ progress.

### Development of extensions to enhance the visual interface

In most cases we did not need to modify the source code of MediaWiki or SMW to develop visual components. Simply modifying the templates as described in the previous section was sufficient. In a limited number of cases, such as to make use of external web services, it was necessary to create SMW extensions (The PHP code of the extensions are available at our web site, http://fantom.gsc.riken.jp/5/suppl/Abugessaisa_et_al_2016/).

One of the extensions we developed embeds a genomic view provided in UCSC Genome Browser (Figure 3A). By calling the extension from the CAGE peak or the EntrezGene template (Figure 2F), SSTAR displays the genomic view in CAGE peak or EntrezGene page. Using this view investigators are able to compare CAGE peaks identified in FANTOM5 with existing genome annotations such as gene models and ENCODE results. Another example is the representation of FF ontology terms. In the T cell (CL: 0000084) term page, e.g. parental terms, such as lymphocyte (CL: 0000542), were shown as text, as in Supplementary Figure S2A. We implemented an MediaWiki extension for an interactive ontology visualization that shows ascendant/descendant ontology terms (Supplementary Figure S2B) by embedding the NCBO’s ontology visualization widget (provided via web services) (Supplementary Figure S2C).

### Data export for genomic data in standard formats

Query tools provide a way to select subsets of the data stored in SSTAR according to specified criteria (biological or statistical), allowing researchers to inspect or download the results for further analysis. Three formats—Resource Description Framework (RDF)/XML, JavaScript Object Notation, and comma-separated values (CSV) plain text—are natively supported by SMW. In particular, the columns in the CSV output can be specified either in the query interface or as parameters of the API. Many file formats in biology are based on tab delimitation (e.g. BED, GFF, SDRF etc.), which is just a variation of the CSV export. Therefore, we extended the system to export query results in a tab-separated values (TSVs) format.

MAGE-tab (25) and ISA-tab (26) are accepted as standard formats for metadata describing experiments in functional genomics and other ‘–omics’ research. Both of them employ SDRF, which requires a specific structure within TSV-formatted files. The TSV export, having flexible columns order, makes it possible to generate sample information in these standard formats. Supplementary Figure S4 shows an example of FANTOM5 metadata, with primary name in the ‘source name’ columns and the other attributes in ‘Characteristics […]’ columns, stored as different properties such as ‘Name’ and ‘Sample species’. We implemented a SSTAR extension for downloading search results in TSV, and we embedded download links in pages for specific tables such as CAGE peaks, FF ontology terms, and samples.

### Performance evaluation

**Figure 4.**
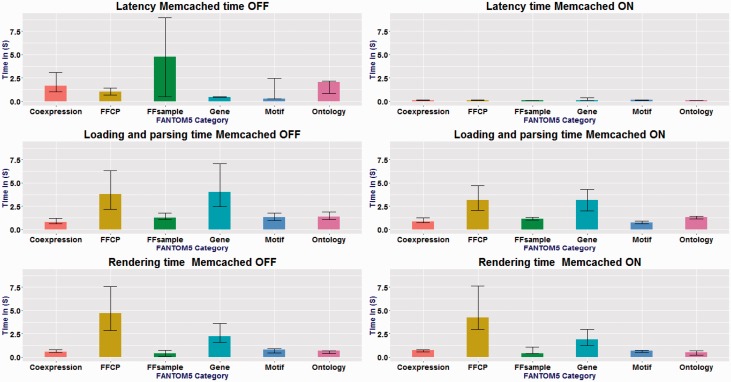
Measurements of page-timing. x-axis shows FANTOM5 categories and the y-axis is the different timing in seconds. Memcached ON and OFF, for the six categories: *n* = 87, *n* = 1003, *n* = 3011, *n* = 52, *n* = 16 and *n* = 10 . The box denotes the median. The bars on each column show the 25 and 75 percentiles. Latency time changed drastically between cache-on and cache-off. No change in the loading and parsing time and rendering time for all categories.

We assessed the performance of SSTAR on the server side and the client side depending on each category of data. Performance varied depending on the page category, with typical durations of less than one second for gene pages and nearly five seconds for sample pages (Figure 4) without memory caching. Use of a memory cache improved the server processing time more than tenfold (i.e. 0.1 s for all page categories; see Figure 4. The durations required on the client side also differ depending on page categories (Figure 4). Detailed results of the performance evaluation are included in the Supplementary Data.

### Comparison to other SMW/MediaWiki-based database systems

SSTAR is not the first application based on SMW in the field of biology. We compared it with other systems using SMW, such as SNPedia (27) and SEQanswer (28) wiki. The statistics summarized in Tables 3 and 4 indicate that SSTAR stores the largest number of pages and semantic properties, with >400 000 pages and >50 000 000 semantic properties, which is four to five times more than the largest database we examined (Pest Information Wiki; http://wiki.pestinfo.org/).

**Table 3.** Number of semantic property values

Category	Semantic property vlaues
Cell ontology	646
Coexpression clusters	4882
EntrezGene	100286
FFCP	721537
FF ontology	5149
FF samples	3605
FF terms	1544
Human disease ontoloy	260
JASPAR motif	113
MCL coexpression mm9	3771
MacroAPE 1083	1080
Motif	691
MotifCluster	204
NonRedundantMotifCluster	210
Novel motif	170
SwissregulonMotif	198
Time courses	36
Uber anatomy ontology	1354

The table shows categories and their corrsponding semantic property values in SSTAR.

**Table 4.** Comparison between SSTAR and other systems using SMW / MediaWiki

System	URL	pages	Number of semantic properties	Semantic property values
FANTOM5 SSTAR	http://fantom.gsc.riken.jp/5/sstar/	415 676	196	54 266 939
ArthropodBase Wiki	http://arthropodgenomes.org/wiki/	9902	171	67 407
Bioinformatics.Org	http://www.bioinformatics.org/wiki/	2378	85	5898
GeneWiki+	http://genewikiplus.org/wiki/	91 379	245	1 978 820
GMOD	http://gmod.org/	3873	54	9 580
MetaBase	http://MetaDatabase.Org/	4618	31	13 286
NeuroLex	http://neurolex.org/	76 374	198	546 389
OpenToxipedia	http://www.opentoxipedia.org/	1280	8	4329
Pest information Wiki	http://wiki.pestinfo.org/wiki/	134 915	41	10 474 99
SNPedia	http://snpedia.com/	111 052	103	4 313 629
SEQanswers wiki	http://SEQanswers.com/wiki/	3338	110	36 623

The number of pages and semantic properties the statistics were collected on the 17 February 2015.

## Conclusion

We discussed the development of SSTAR for the FANTOM5 project. SSTAR facilitates managing and publishing of large sets of biological and genomic data and remains adaptable to the changing needs of ongoing research activities. The system allows investigators with different backgrounds to explore the FANTOM5 heterogeneous resource. This approach has been effective for our ongoing and large-scale research project, where novel analysis strategies and findings have been be provided as research progressed. We were able to expand functionalities, such as enhancement of graphical representation and management of data for genomic standards, by developing small extensions without building, testing, debugging an entirely new system from scratch. We also examined the scalability of the system and found that a large number of properties can be handled in an acceptable response time. This suggests that the framework is applicable even when working with data larger than the existing biological databases that rely on SMW. To conclude, the MediaWiki/SMW framework has enabled us to overcome the challenges we experienced to create a scalable system with the capabilities to handle FANTOM5 data sets and the associated analysis results. Other biological database developers could also implement their own database with SMW, storing their data as wiki texts in the system with flexible adaptation of graphical components in a flexible ‘wiki’ manner and extension systems. Our use case in this report delineated successful design principles and configurations as well as the scalability required for genomic research.

## Availability

FANTOM5 SSTAR database system is accessible from

(i) http://fantom.gsc.riken.jp/5/sstar/

(ii) The PHP code for the SSTAR extensions, and supplemental data about our performance evaluations are available at our web site, http://fantom.gsc.riken.jp/5/suppl/Abugessaisa_et_al_2016/.

## Supplementary data

Supplementary data are available at *Database* Online.

Supplementary Data
