# Supplementary material for: FANTOM5 transcriptome catalog of cellular states based on Semantic MediaWiki
Source: Database (Oxford). 2016 Jul 9;2016:baw105. doi: 10.1093/database/baw105 (PMC4940433; doi:10.1093/database/baw105)
Supplement: Supplementary Data [file supp_2016_baw105_index.html]

FANTOM5 transcriptome catalog of cellular states based on Semantic MediaWiki — Supplementary Data 

# FANTOM5 transcriptome catalog of cellular states based on Semantic MediaWiki

## Supplementary Data

files

- Supplementary Data - zip file
