# Supplementary material for: FANTOM5 transcriptome catalog of cellular states based on Semantic MediaWiki
Source: Database (Oxford). 2016 Jul 9;2016:baw105. doi: 10.1093/database/baw105 (PMC4940433; doi:10.1093/database/baw105)
Supplement: Supplementary Data [file supp_baw105_suppl_data.zip › Supp_figure_3.pdf]

```
function collectdata(){
data = [];
                    for (i2=1, len2=oTable.fnSettings().aoColumns.length; i2<len2; i2++){
                              var plots = [];
                              for (i1=2, len1=$('#gene_exptable tr').length; i1<len1; i1++) {
                                        plots.push([i1,$("#gene_exptable
tr")[i1].cells[i2].innerHTML]);
                              }
                              data.push({"label":
$(oTable.fnSettings().aoColumns[i2].sTitle).text(), "data": plots});
                    }
slist = [];
                              for (i1=2, len1=$('#gene_exptable tr').length; i1<len1; i1++) {
                                        slist.push($("#gene_exptable
tr")[i1].cells[0].innerHTML);
                              }
}
function drawchart(){

plotdata = [];

$("#gene_exptable_chart_chkbox").find("input:checked").each(function () {
                    var key = $(this).attr("name");
                    plotdata.push(data[$(this).attr("name")]);
});

var plot1 = $.plot($("#gene_exptable_chart"), plotdata, {
          yaxis : { axisLabel : 'TPM' },
                    lines : { show : false },
                    points : { show : true },
          grid: { hoverable: true, clickable: true },
                    xaxis : { tickDecimals : 0, show: false      },
          legend: { show: false }
});
```

Figure S3 JavaScript for graphical and interactive exploration of TSSs expression
Custom JavaScript code has been added to reflect data in (Figure 3B) and (Figure 3C).
