# Supplementary material for: FANTOM5 transcriptome catalog of cellular states based on Semantic MediaWiki
Source: Database (Oxford). 2016 Jul 9;2016:baw105. doi: 10.1093/database/baw105 (PMC4940433; doi:10.1093/database/baw105)
Supplement: Supplementary Data [file supp_baw105_suppl_data.zip › Supp_figure_4.pdf]

(A)

```
{{#ask:[[Category: FF Samples]]
|?#
|?Name=Source Name
|?Sample species
|?Sample sex=Characteristics [sex]
|?Rna extraction protocol=RNA extraction protocol
|?Id=Sample Name
}}
```

(B)

	Source Name	Sample species	Characteristics [sex]	RNA extraction protocol	Sample Name
10000-101A1	Clontech Human Universal Reference Total RNA, pool1	Human (Homo sapiens)	mixed	OP-RNA-extraction-totalRNA-TRIzol-isopropanol-v1.0	FF:10000-101A1
10001-101A3	Clontech Mouse Universal Reference Total RNA, pool1	Mouse (Mus musculus)	mixed	OP-RNA-extraction-totalRNA-TRIzol-isopropanol-v1.0	FF:10001-101A3
10002-101A5	SABiosciences XpressRef Human Universal Total RNA, pool1	Human (Homo sapiens)	mixed	OP-RNA-extraction-totalRNA-TRIzol-isopropanol-v1.0	FF:10002-101A5
10003-101A7	SABiosciences XpressRef Mouse Universal Total RNA, pool1	Mouse (Mus musculus)	mixed	OP-RNA-extraction-totalRNA-TRIzol-isopropanol-v1.0	FF:10003-101A7
10004-101A9	chicken embryo whole body, day10 (HH37)	Chicken (Gallus gallus)	unknown	OP-RNA-extraction-totalRNA-TRIzol-isopropanol-v1.0	FF:10004-101A9
10006-101B2	Universal RNA - Dog Normal Tissues Biochain, pool1	Dog (Canis lupus familaris)	mixed	OP-RNA-extraction-totalRNA-TRIzol-isopropanol-v1.0	FF:10006-101B2
10007-101B4	Universal RNA - Human Normal Tissues Biochain, pool1	Human (Homo sapiens)	mixed	OP-RNA-extraction-totalRNA-TRIzol-isopropanol-v1.0	FF:10007-101B4
10008-101B6	Universal RNA - Mouse Normal Tissues Biochain, pool1	Mouse (Mus musculus)	mixed	OP-RNA-extraction-totalRNA-TRIzol-isopropanol-v1.0	FF:10008-101B6
10009-101B8	Universal RNA - Rat Normal Tissues Biochain, pool1	Rat (Rattus rattus)	mixed	OP-RNA-extraction-totalRNA-TRIzol-isopropanol-v1.0	FF:10009-101B8
10010-101C1	adipose tissue, adult, pool1	Human (Homo sapiens)	mixed	OP-RNA-extraction-totalRNA-ToTALLY-RNA-v1.0	FF:10010-101C1

Fig S4. Sample attributes list generated with semantic query
Semantic query (A) is used to list and export information regarding biological sample attributes (B).
