# Supplementary material for: FANTOM5 transcriptome catalog of cellular states based on Semantic MediaWiki
Source: Database (Oxford). 2016 Jul 9;2016:baw105. doi: 10.1093/database/baw105 (PMC4940433; doi:10.1093/database/baw105)
Supplement: Supplementary Data [file supp_baw105_suppl_data.zip › Supplementary information.docx]

# Supplementary information

## Supplementary Results

### Performance evaluation result

In addition to the functionalities for users and the flexibility in implementation, performance of the system (that is, how quickly the system can interact with users) is an important aspect. Here we assess the performance of SSTAR on the server side and the client side depending on each category of data, since size and complexity of the data differ considerably across categories. We also assess the impact of memory caching, which is employed in the configuration of the SSTAR server.

In measuring performance, we focused on the time interval required to display all graphical elements of a page after a user’s action (the orange and green parts in **Figure 2B**). The duration can be divided into server-side processing time (orange arrow) and client-side parsing and rendering time (green laptop). The server processing time, measured as latency, is the duration required to generate page contents (here, several MediaWiki templates) on the server and send it to the client after a user requests pages in SSTAR. The server settings has also been evaluated, mainly server side memory caching (Memcached, <http://www.mediawiki.org/wiki/Manual:Configuration_settings#Memcached_settings>) has been measured.

The result from the performance test is illustrated and summarized in the **Figure 4** and the summary statistics is available at our web site, http://fantom.gsc.riken.jp/5/suppl/Abugessaisa_et_al_2016/. The response time to individual operations is relatively short, less than ten seconds for most pages. The sample page requires a series of semantic queries, such as to show associated TFBS (DNA) motifs, relative expression of transcription factors, and co-expression clusters, caching results of their queries likely contributes to the substantial reduction in response time.

In contrast to the processing time at the server side, where FF samples take the longest time, both loading/parsing and rendering time on the client side take the longest in the Gene and CAGE peaks (FFCP) categories. Since both of these categories show expression levels across nearly one thousand samples graphically by relying on a JavaScript library, optimization of this process could improve performance.

As shown in (**Figure 4**), we noticed many outliers in the client side performance for the FFCP and Gene categories. For 3011 sampled pages of FFCP the median is 3.15 seconds; we observed an outlier with loading and parsing time of 38.9 seconds. For the Gene category (n=1003 pages), the median time is 3.16 seconds with an observed outlier for pages that took 30.3 seconds. The outliers in the loading and parsing time are associated with one of the following factors: (1) network traffic and/or (2) the FFCP and Gene templates are using UCSC API to retrieve the UCSC genome browser view of the CAGE peaks and annotated Genes.

Given that it takes negligible time on the server side when using memory cache, the total time for a user to receive the final visualization is equivalent to the total time of loading, parsing, and rendering time, which is less than 10 seconds for 75% of the pages.

## Supplementary Method

### Performance testing

SSTAR’s pages were randomly selected for test, to measure distribution of required time per page. (The full list of the pages is available at our web site, http://fantom.gsc.riken.jp/5/suppl/Abugessaisa_et_al_2016/). Each of the durations was measured using Firebug (<http://getfirebug.com/>, a Firefox add-on) which monitors the time required for each process and produces a single file in HTTP Archive format (see <https://dvcs.w3.org/hg/webperf/raw-file/tip/specs/HAR/Overview.html>) consisting of logged data as well as transferred data. Latency (the server response time) is recorded in the log data as waiting time, loading and parsing time is calculated as (OnContentLoad time **–** Latency time), and rendering time is calculated as (OnLoad time **–** OnContentLoad time). iMacro (also an extension of Firefox, <http://imacros.net/>) was used to automate iteration of the monitoring. The resulting collection of .har files were parsed and processed using a Python program to generate the summary of the test results. All used iMacros scripts and the processed result files are available at our web site, http://fantom.gsc.riken.jp/5/suppl/Abugessaisa_et_al_2016/.

The following hardware was used for this performance testing: local hard disk drive 909 GB (RAID1), Memory 72 GB in total, 2 CPUs of Intel® Xeon® CPU E5540 at 2.53 GHz (16 Cores in total), which is an SSTAR production environment. The software environment consists of Apache 2.2, Centos x86 64, MySQL version 5.5.33 and PHP 5.4.7, MediaWiki version 1.19.2, and Semantic MediaWiki version 1.7.1. For the client we used a machine with Windows 8.1 Pro and installed memory of 8.00 GB running with Intel® Core^TM^ i7-4600U CPU at 2.10 GHz and Firefox 40.0.3.
