# Supplementary material for: FANTOM5 transcriptome catalog of cellular states based on Semantic MediaWiki
Source: Database (Oxford). 2016 Jul 9;2016:baw105. doi: 10.1093/database/baw105 (PMC4940433; doi:10.1093/database/baw105)
Supplement: Supplementary Data [file supp_baw105_suppl_data.zip › Supp_figure_1.pptx]

## Slide 1
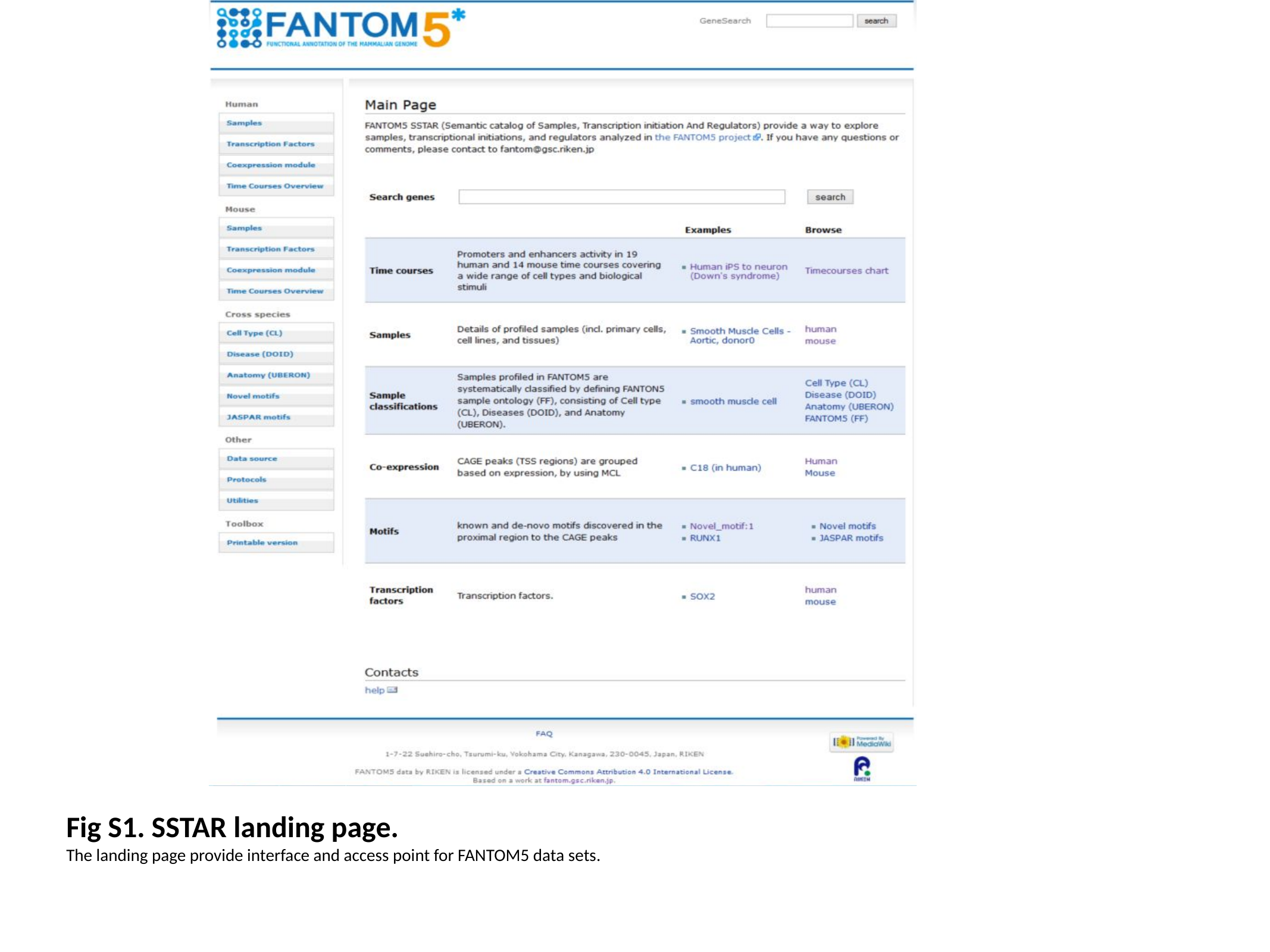

Fig S1. SSTAR landing page.
The landing page provide interface and access point for FANTOM5 data sets.
