# Supplementary material for: FANTOM5 transcriptome catalog of cellular states based on Semantic MediaWiki
Source: Database (Oxford). 2016 Jul 9;2016:baw105. doi: 10.1093/database/baw105 (PMC4940433; doi:10.1093/database/baw105)
Supplement: Supplementary Data [file supp_baw105_suppl_data.zip › Supp_figure_2.pptx]

## Slide 1
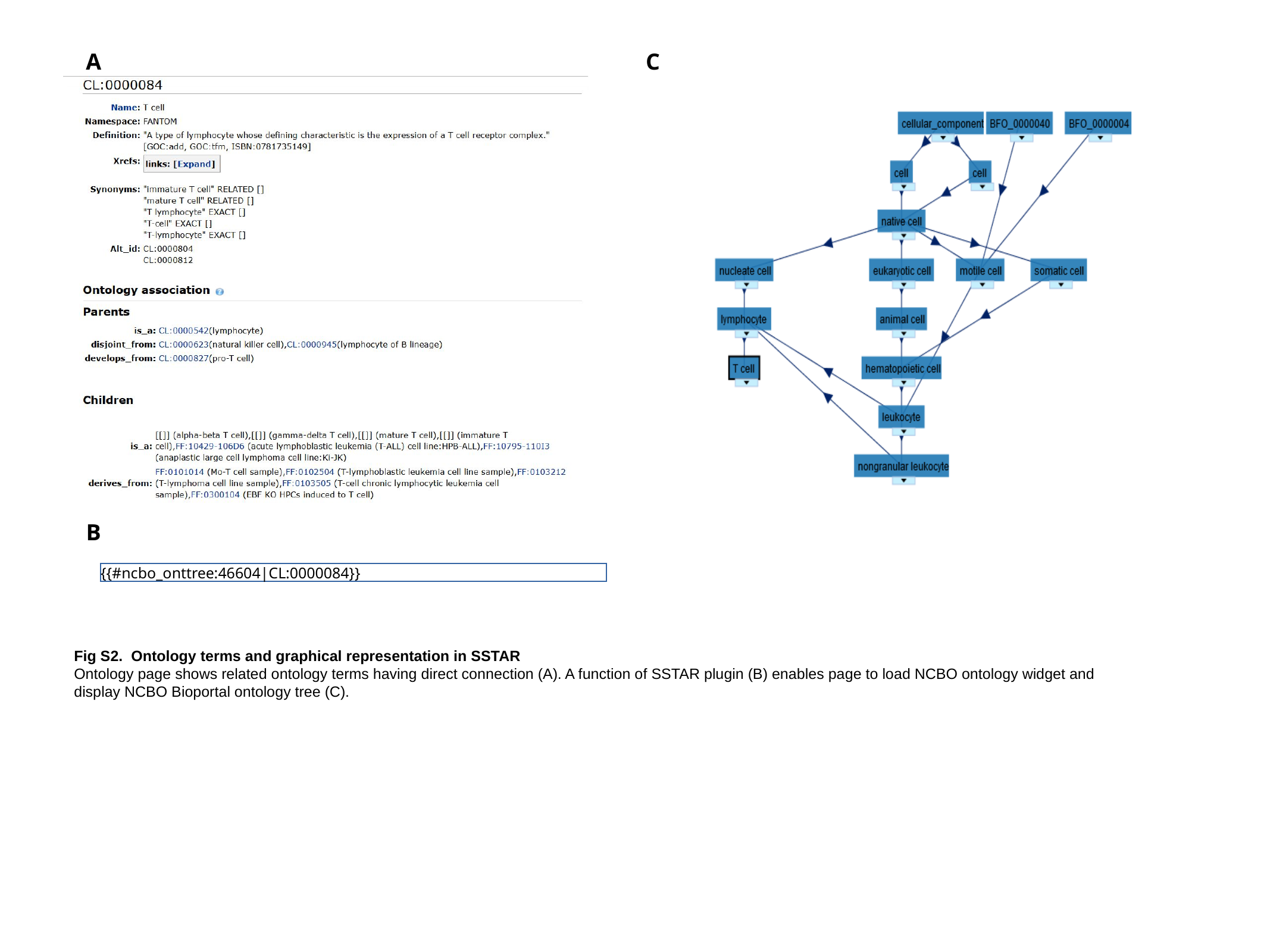

A
C
B
{{#ncbo_onttree:46604|CL:0000084}}
Fig S2. Ontology terms and graphical representation in SSTAR
Ontology page shows related ontology terms having direct connection (A). A function of SSTAR plugin (B) enables page to load NCBO ontology widget and display NCBO Bioportal ontology tree (C).
